# *Invaders Exposed:* Understanding and Targeting Tumor Cell Invasion in Diffuse Intrinsic Pontine Glioma

**DOI:** 10.3389/fonc.2020.00092

**Published:** 2020-02-07

**Authors:** T. A. Kluiver, M. Alieva, D. G. van Vuurden, Ellen J. Wehrens, Anne C. Rios

**Affiliations:** ^1^Princess Máxima Center for Pediatric Oncology, Utrecht, Netherlands; ^2^Department of Cancer Research, Oncode Institute, Hubrecht Institute, KNAW Utrecht, Utrecht, Netherlands; ^3^Cancer Genomics Center, Utrecht, Netherlands

**Keywords:** diffuse intrinsic pontine glioma (DIPG), invasion, driver mutations, tumor subclones, microenvironment, therapeutic targeting

## Abstract

Diffuse Intrinsic Pontine Glioma (DIPG) is a rare, highly aggressive pediatric brain tumor that originates in the pons. DIPG is untreatable and universally fatal, with a median life expectancy of less than a year. Resection is not an option, due to the anatomical location of the tumor, radiotherapy has limited effect and no chemotherapeutic or targeted treatment approach has proven to be successful. This poor prognosis is partly attributed to the tumor's highly infiltrative diffuse and invasive spread. Thus, targeting the invasive behavior of DIPG has the potential to be of therapeutic value. In order to target DIPG invasion successfully, detailed mechanistic knowledge on the underlying drivers is required. Here, we review both DIPG tumor cell's intrinsic molecular processes and extrinsic environmental factors contributing to DIPG invasion. Importantly, DIPG represents a heterogenous disease and through advances in whole-genome sequencing, different subtypes of disease based on underlying driver mutations are now being recognized. Recent evidence also demonstrates intra-tumor heterogeneity in terms of invasiveness and implies that highly infiltrative tumor subclones can enhance the migratory behavior of neighboring cells. This might partially be mediated by “tumor microtubes,” long membranous extensions through which tumor cells connect and communicate, as well as through the secretion of extracellular vesicles. Some of the described processes involved in invasion are already being targeted in clinical trials. However, more research into the mechanisms of DIPG invasion is urgently needed and might result in the development of an effective therapy for children suffering from this devastating disease. We discuss the implications of newly discovered invasive mechanisms for therapeutic targeting and the challenges therapy development face in light of disease in the developing brain.

## Introduction

Diffuse intrinsic pontine glioma (DIPG) is a rare, highly aggressive pediatric brain tumor that originates from the pons. DIPG is untreatable and universally fatal, making it the leading cause of pediatric brain tumor-related deaths (1). Clinical presentation occurs between 0 and 26.8 years of age, with a median age of 6.8 years. Median overall survival following initial diagnosis is 11 months and 2-year survival rate is <10% (2). One key characteristic of DIPG, next to its origin in the pons, is the tumor's highly diffuse and invasive growth. Although in a survival prediction model, DIPG spread to the medulla and midbrain was not a significant prognostic value (3), in a different study extrapontine extension was shown to be more common in short-term compared to long-term survivors (2). This impact on DIPG prognosis can be due to the ability of invading DIPG cells to distort, displace, and destroy nerve fiber tracts (4). For instance, specific symptoms like impairment of gait, coordination, and speech, are thought to be associated with invasion of tumor cells to the adjacent cerebellum (5). It has furthermore been suggested that combating invasion could potentially help improve both quality of life and survival, and reduce serious secondary neurological effects (6, 7). Although local progression is still seen as the main problem in DIPG, these findings clearly indicate the potential role of invasion to worse prognosis. Moreover, because of the tumor's highly diffuse invasive phenotype, any local treatment will only have limited benefit. Indeed, focal radiation therapy to the pons, which remains standard of care, improves life expectancy by only a few months (8, 9). This very limited short-term efficacy remains one of the major challenges with DIPG and correlates with the widespread dissemination to extra-pontine regions (10). Furthermore, despite a fair amount of clinical trials, including several testing more targeted treatments, there has been no clear improvement in the prognosis of DIPG since the introduction of radiotherapy (11, 12). This has been partially attributed to the drug selection process for DIPG clinical trials being guided by molecular targets identified in its adult counterpart glioblastoma multiforme (GBM) (13). However, significant molecular and cellular differences have now been demonstrated between the two diseases (14), highlighting a critical need for new therapies specific to DIPG. Due to its highly infiltrative nature, targeting the invasive behavior of DIPG has the potential to improve life expectancy. In this review, we will outline the current knowledge on DIPG invasion patterns, the molecular and cellular mechanisms that underlie this process, and discuss the potential of targeting invasion for therapy.

## Tumor Cell-Intrinsic Molecular Alterations Can Contribute to DIPG Invasion

### DIPG Specific Driver Mutations Have Been Identified That Could Promote Invasion

**Figure 1:**
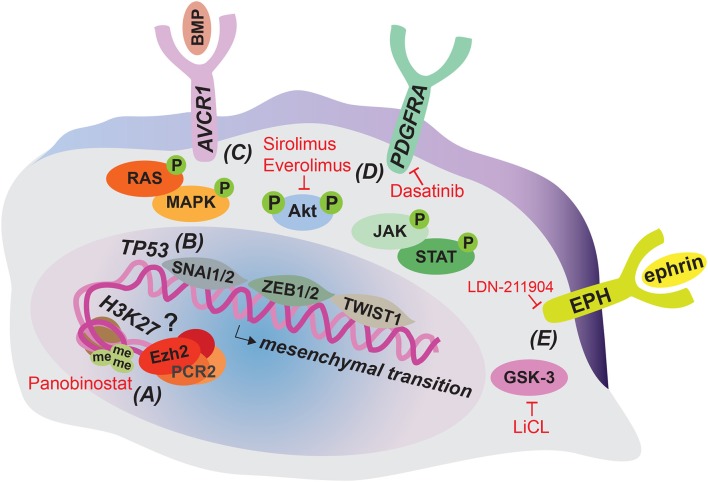
Tumor cell-intrinsic alterations that can contribute to DIPG invasion and strategies for their therapeutic targeting. The majority of DIPG tumors harbor a H3K27M mutation resulting in epigenetic dysregulation that might contribute to invasion and can be targeted therapeutically by Panobinostat **(A)**. Loss of function mutations in *TP53* are common in DIPG and can enhance migratory cell behavior by inducing the mesenchymal transcription factor SNAI1 **(B)**. Gain of function mutations in *AVCR1* **(C)** and *PDGFRA* **(D)** activate downstream RAS/MAPK, PI3K/Akt, and JAK/STAT signaling, which can induce invasion associated mesenchymal transition through SNAI1/2, ZEB1/2, and TWIST1 transcription factors. Enhanced PI3K/Akt signaling can be therapeutically targeted by Sirolimus and Everolimus and PDGFRA signaling by Dasatinib. EPH-ephrin signaling and GSK-3 have been implicated in migratory behavior in DIPG cell cultures and can be targeted therapeutically by LDN-211904 and LiCL, respectively **(E)**.

Tumor cell invasion is a complex process regulated by numerous intrinsic genetic, as well as extrinsic environmental factors. Since the recent introduction of biopsies and autopsies in DIPG, efforts have been made to characterize the genomic landscape of this disease (15, 16). These studies have elucidated recurring mutations, which might intrinsically contribute to the tumor's diffuse spread, although this still needs to be addressed experimentally. The majority of DIPG cases (~80%) carry a specific point mutation in one of the histone 3 genes; H3.3 (*H3F3A*) or H3.1 (*HIST1H3B*), that results in the substitution of lysine 27 on the amino-terminal tail for a methionine (H3K27M) (15, 17, 18). Interestingly, mutations in *H3F3A* resulted in worse prognosis than mutations in *HIST1H3B* (19). Identification of this recurrent mutation has led the World Health Organization (WHO) to reclassify this larger subset of DIPG into the new entity of “diffuse midline glioma, H3K27M mutant” (20). Other recurrent genomic alterations or amplifications are found in tumor protein p53 (*TP53*) (16, 18), platelet-derived growth factor receptor alpha (*PDGFRA*) (14, 21), and activin A receptor type I (*ACVR1*) (15, 18, 22, 23), along with many more at lower frequencies more comprehensively described by Mackay et al. (16), including *ATRX* (24). Although very little is known about how these mutations impact DIPG invasion, some data suggest that these intrinsic alterations can contribute to tumor cell migratory behavior (Figure 1).

#### H3K27M

The histone H3 K27 residue is subject to post-translational modification, including acetylation and methylation. Lysine acetylation is associated with active transcription, while methylation results in repressive marks and reduced gene expression. Enhancer of zeste homolog 2 (EZH2), the catalytic subunit of the Polycomb-repressive complex 2 (PRC2) is responsible for trimethylation of the K27 residue (H3K27me3) (25). The DIPG associated mutation H3K27M prevents lysine 27 from being methylated. In addition, H3K27M peptide inhibits PCR2 activity by binding to the EZH2 subunit (26). Overall, this results in a global reduction in H3K27me3 levels and thus overall gene repression, which could contribute to a less differentiated cellular state. Indeed, using single-cell RNA sequencing, Filbin et al. show that pediatric diffuse midline gliomas that carry the H3K27M mutation primarily contain cells that resemble oligodendrocyte precursor cells, whereas more differentiated malignant cells are scarce. This overrepresentation of oligodendrocyte precursor-like cells could contribute to tumor progression, since these cells were shown to have a more tumor-propagating and proliferative phenotype compared to their more differentiated counterparts (27). Despite the global reduction in H3K27me3, surprisingly, H3K27me3 levels are retained or even increased at several gene loci, including those of several tumor-suppression genes and genes involved in immune recognition. Therefore, this repression could contribute to tumorigenesis as well (28, 29). In a follow-up report, Zhang and colleagues solve the seemingly paradoxical finding of enhanced H3K27me3 at certain gene loci, by showing that the PCR2 complex is preferentially sequestered to permissive enhancers with relative low levels of H3K27M mutant proteins compared to other loci, which minimally affects PCR2 activity at these sites (30). This epigenetic dysregulation shared by the majority of DIPG tumors can potentially also affect genes involved in invasion. Indeed, increased expression of the extracellular matrix glycoprotein Tenascin-C (TNC) correlates with the H3K27M mutation in DIPG and experimental manipulation of TNC expression levels reveals a direct role of TNC in DIPG cell invasive behavior, at least *in vitro* (31). In addition, EZH2 has been shown to be required for the proper migration of neurons into the pons during development (32) and upregulation of EZH2 has been implicated in invasion in a variety of cancers (33–36). Therefore, although it remains to be determined how alterations in EZH2 specifically affect DIPG invasion, it is conceivable that altered EZH2 activity and subsequent H3K27me3 levels impact DIPG invasion (Figure 1A).

#### TP53

*TP53* is one of the most commonly mutated cancer genes, and loss of function has been shown to drive invasion by promoting integrin recycling and causing accumulation of the zinc finger protein snail family transcriptional repressor 1 (*SNAI1*), a central player in epithelial-to-mesenchymal transition (EMT) (37, 38). EMT involves the loss of cell-cell adhesion and polarity in epithelial cells, and the acquisition of a more mesenchymal, invasive phenotype (39). Indeed, this process has been heavily associated with malignant invasive and migratory behavior for many types of cancer (40). In DIPG, mesenchymal transition of glial cells, including the upregulation of mesenchymal related transcription factors and the induction of a mesenchymal phenotype could contribute to tumor invasion as well (41). Loss of TP53 is relatively common in DIPG (17) and, although not directly studied, could contribute to its invasive nature by promoting mesenchymal transition though SNAI1 (Figure 1B). In line with this, EMT-associated pathways and transcription factors have been found activated in DIPG (41). Moreover, in adult GBM a higher incidence of *TP53* mutation was observed in tumors with a diffuse compared to non-diffuse classification (42).

#### ACVR1

The transforming growth factor-beta superfamily member *ACVR1* is mutated in DIPG with intermediate frequency (varying from 20 to 32% of cases studied) (15, 18, 22, 23). Also known as *ALK2, ACVR1* encodes a bone morphogenetic protein (BMP) type 1 receptor. In affected cases of DIPG, *ACVR1* carries constitutively activating mutations that result in increased BMP signaling (22). Activation of the BMP pathway induces the accumulation of SMAD proteins in the nucleus, which can subsequently activate EMT-related transcription factors such as SNAI1/2 and zinc finger E-box-binding homeobox 1/2 (ZEB1/2) (43). Moreover, activation of ACVR1 can lead to phosphoinositide 3-kinase/protein kinase B (PI3K/Akt), rat sarcoma/mitogen-activated protein kinase (RAS/MAPK) and Janus kinase/signal transducer and activator of transcription (JAK/STAT) signaling (43–45). This can induce mesenchymal transition and contribute to invasion, as all three of these pathways are known to activate EMT-related transcription factors, including twist-related protein 1 (TWIST1) (Figure 1C). P13K/Akt stabilizes SNAI1/2, induces transcription of ZEB1 and phosphorylates TWIST1, resulting in its activation (46–48). Moreover, a recent study shows that in particular ACVR1 R206H mutants induce a mesenchymal phenotype by STAT3 signaling activation and associate with shorter survival (49). This seems to indicate that distinct ACVR1 mutations have differential effects on DIPG tumor progression as other non-R206H mutants have been associated with longer survival rate (23).

Next to activating mutations in *ACVR1*, the PI3K/Akt pathway can also be upregulated due to mutations in *PI3KCA* and *PIK3RI*, encoding subunits of PI3K, which occur in 10–15% of patients (50, 51). In addition, BMP induced RAS/MAPK signaling increases transcription of SNAI1/2 and phosphorylates TWIST1 (52, 53) and JAK/STAT activation up-regulates all three EMT related transcription factors (54–56). Indeed, when brainstem progenitor cells were transduced with DIPG associated mutations in *ACVR1*, SMAD phosphorylation, STAT3 signaling, and mesenchymal marker expression were induced (49). In addition, a meeting abstract reports that inhibition of the JAK/STAT component STAT3 in human DIPG cell lines results in a reduced migratory phenotype (57). Although these data still await full publication, this would confirm a direct role for JAK/STAT signaling in DIPG invasion.

#### PDGFRA

Copy number gain in *PDGFRA* is a relatively frequent event in DIPG, occurring in ~30% of patients (14, 21). So far, alterations in *PDGFRA* provide the strongest link to DIPG invasive behavior. This gene encodes a receptor tyrosine kinase that when activated can induce downstream PI3K/Akt, RAS/MAPK, and JAK/STAT signaling, which as described above can promote mesenchymal transition by inducing EMT-related transcription factors (41), including ZEB1 (Figure 1D). Moreover, PDGFRA and ZEB1 cooperate to induce mesenchymal transition in adult glioma (58), further stressing the link between PDGFRA and mesenchymal transition. Most importantly, direct proof for a role of PDGFRA in DIPG invasion comes from a study that developed an elegant *in utero* electroporation model for DIPG (59). In this model, combined introduction of H3.3K27M and knockdown of both ATRX and p53 in the developing mid- or forebrain induced DIPG-like tumors. When additional overexpression of PDGFRA was introduced, the resulting tumors were significantly more invasive, providing a direct link between *PDGFRA* gain of function and DIPG invasion.

In conclusion, with the exception of *PDGFRA* described above, for the other DIPG driver mutations a direct involvement in invasive behavior still requires experimental confirmation. However, it is striking that the majority of recurrent mutations found in DIPG promote pathways and transcription factors that enhance mesenchymal transition and in doing so have been implicated in invasion for other types of cancer. This not only reveals a promising opportunity to directly study the impact of these mutations on DIPG invasion, but also further confirms the importance of tumor cell invasion in DIPG progression.

### Other Cell-Intrinsic Molecular Pathways Playing a Role in DIPG Invasion

Additional molecular pathways have been described that could play a role in DIPG invasion, without being directly linked to specific driver mutations (Figure 1E). However, it should be kept in mind that activation of these pathways could be the result of the overall H3K27M induced shift in epigenetic regulation. A number of ephrin and EPH receptors were found to be associated with super enhancer regions in patient-derived DIPG cell cultures. When this pathway was inhibited using a selective inhibitor of forward EPH receptor signaling, invasion was reduced in a 3D migration assay (60). This is consistent with the reported role of the EPH-ephrin pathway in adult GBM invasion (51, 61). In addition, inhibition of glycogen synthase kinase 3 (GSK-3), which is associated with cell migration by regulating cytoskeleton and cell-matrix interactions (62), also inhibits invasiveness in a multi-dimensional migration assay with a patient-derived DIPG cell line (63).

While more and more molecular and genetic pathways underlying DIPG are being described, it appears that we still have barely scratched the surface of the intrinsic molecular mechanisms contributing to DIPG invasion. With recent insights into the underlying driver mutations and molecular pathways affected in DIPG, it will be essential to generate more data on how these intrinsic features contribute to DIPG invasion, especially given the prominent role of invasion in the poor prognosis of this disease.

## Intra-Tumor Cooperation Between DIPG Subclones Promotes Invasion

**Figure 2:**
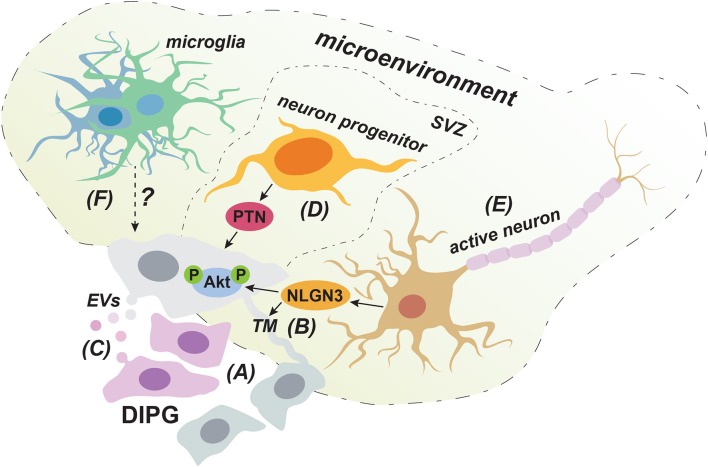
Cell-extrinsic factors that can promote the invasive behavior of DIPG cells. DIPG tumors consist of a cooperative network of cellular subclones in which highly migratory clones can enhance the invasive behavior of less motile cells **(A)**. Cellular communication via both TMs **(B)** and EVs **(C)** could play a role in this clonal synergy in invasive behavior. Neuron progenitor cells promote DIPG invasion into the SVZ through the production of PTN **(D)**. Secretion of NLGN3 by activated neurons could contribute to DIPG invasion by inducing the formation of TMs and by activating the PI3K/Akt pathway **(E)**. The immune cell infiltrate in DIPG mainly consist of microglia/macrophages with a low inflammatory phenotype and it is still unclear how this impacts invasion **(F)**.

Next to genomic and molecular alterations intrinsic to the tumor cell, signals from neighboring tumor cells or the non-tumor microenvironment can influence a tumor cell's invasive behavior (Figure 2). While great progress has been made in identifying underlying molecular pathways and driver mutations of DIPG through transcriptomic, genomic and epigenomic approaches, the majority of this work was done using bulk sequencing technologies. However, DIPG tumors are highly heterogeneous and consist of multiple distinct tumor subclones (64, 65). This can significantly impact DIPG's invasive nature, as recent evidence suggests that distinct subclones within a DIPG tumor can engage in cooperative signaling to promote tumor cell invasion. An elegant study shows that while genetically and phenotypically diverse DIPG subclones stably coexist and invade with different efficiencies *in vitro* and *in vivo*, they invade more efficiently when co-cultured or transplanted together (65). Highly invasive tumor cell subclones conferred a clear enhancement of invasive and migratory behavior on their poorly motile counterparts. Genetic analysis identified a specifically invasive subclone with an inactivating mutation in lysine methyltransferase 5B (*KMT5B*) that conferred invasive behavior on neighboring cells, presumably through expression of chemokines, such as chemokine ligand 2 (CXCL2). Thus, in DIPG clonal synergy exists with different tumor subclones activating each other to invade more aggressively (65) (Figure 2A). This implies that disrupting this cooperative network of tumor subclones has the potential to reduce the invasiveness of DIPG tumors. Future efforts should therefore be directed at improving our understanding of how these subclones communicate, which next to secreted factors, like CXCL2, could potentially be achieved through direct physical interactions and exchange of tumor vesicles.

### Tumor Microtubes Could Provide Physical Connections Between DIPG Cells

Clonal synergy in DIPG could be achieved through direct physical connections established though so-called tumor microtubes (TMs) (Figure 2B). These long membranous tubes have been described in adult GBM to provide a physical connection between neighboring tumor cells through gap junctions and can be used for invasion (66). The TM-connected cellular network allows tumor cells to communicate over long distances though intracellular calcium waves and was shown to confer resistance to radiation therapy, as TM-connected cells were largely protected from cell death (66). Given its very poor responsiveness to radiotherapy, a similar resistance mechanism might apply to DIPG. Indeed, the presence of TMs in primary human DIPG tissue has recently been demonstrated (67). Interestingly, according to a yet to be published meeting abstract, the synaptic protein Neuroligin-3 (NLGN3) is sufficient to induce the formation of TM-like structures in DIPG cell cultures (68). This is in line with NLGN3 inducing the expression of growth-associated protein 43 (GAP-43), which has been shown to play a role in the formation of TMs in GBM (66). NLGN3 can be derived from neurons (69) and it is certainly conceivable that neurons can induce TMs, as gap-junction mediated connections between cells in the central nervous system are common and thought to connect neurons, astrocytes, microglia, and oligodendrocytes (70, 71). This implies that DIPG tumor cells could potentially also form connections to healthy brain cells, especially at the invasive tumor front, which could provide them with routes for invading distant sites. Indeed, although physical connections with non-neoplastic cell were not specifically investigated, in adult GBM long cellular protrusions similar to TMs were observed to infiltrate normal brain tissue at the invasive tumor front (66). While the direct involvement of TMs in DIPG invasion remains to be determined, further investigating this intriguing possibility might reveal new directions for therapy development.

### Extracellular Vesicles Can Contribute to Intratumor Communication

Another means for cellular communication that could contribute to clonal cooperation in invasive behavior might be through the secretion of extracellular vesicles (EVs) (Figure 2C), a process known to be hijacked and dysregulated in cancer. Although so far only a meeting report describes the secretion of EVs in DIPG (72), the existence of EVs has been well-established in GBM. EVs can contain a wide range of cargo varying from membrane proteins, cytolytic proteins and lipids to genetic material, such as DNA, mRNA, and microRNAs (73). Notably, GBM derived EVs seem to match the tumor's mutational background, for instance regarding the presence of mRNA for the oncogenic mutant slice variant of the epidermal growth factor receptor III (*EGFR vIII*) (74, 75). Therefore, given the acquired insight into the genetic and molecular alterations underlying DIPG and the presence of tumor subclones, it will be interesting to see whether the content of DIPG derived EVs reflect the cell's mutational background and whether uptake of these EVs can affect the migratory behavior of clones that carry different oncogenic alterations. Importantly, EVs derived from a GBM cell line were shown to enhance the *in vitro* migratory capacities of GBM cells following pre-incubation (76). In addition, in other tumor types, such as breast cancer, uptake of EVs secreted by highly migratory cells has shown to enhance the invasive capacity of less migratory cells *in vivo* (77). Therefore, in addition to enhancing invasion by modulating non-neoplastic cells in the tumor microenvironment (78), EVs can directly impact the migratory behavior of recipient tumor cells, which might represent another means for clonal synergy in DIPG invasion.

## DIPG Preferential Invasion Patterns and First Evidence for Involvement of the Microenvironment

Knowing the preferred routes and dissemination sites of invading tumor cells can provide further cues into both tumor specific and environmental factors involved in invasive behavior. However, knowledge on DIPG invasion patterns is limited and mainly derived from autopsy material, as *in vivo* studies of cellular behavior are still rare, due to a limited availability of suitable experimental models (79). Caretti et al. examined autopsy material from 16 patients and observed that all patients presented with malignant cells in the pons, in line with the presumed origin of DIPG in this specific location (10). However, tumor cells can spread to other parts of the brainstem; medulla and midbrain, adjacent regions, like thalamus and cerebellum and even distant locations, such as the cerebrum (10, 80). At least in some cases, metastatic disease of the subventricular frontal lobe preceded pontine recurrence and contributed to decreased survival (10). Moreover, although only examined in a small number of patients, Caretti et al. observed metastases in the spinal cord in 2 out of 3 patients (10). In addition, in specific cases metastases in the peritoneal cavity have been found following the placement of a ventriculoperitoneal shunt, possibly from tumor seeding in the cerebrospinal fluid (81, 82). In general, however, it appears that DIPG is largely limited to the central nervous system, although it is unclear whether the tumor cannot invade other tissues or whether the disease is simply lethal before it has the opportunity to spread further.

Phylogenetic modeling of DIPG evolution through genome sequencing data derived from different locations within a single tumor, demonstrated not a single clonal expansion, but multiple co-dominant subclonal populations with differential infiltration patterns (65). A limited number of main driver mutations is universally present in tumor cells, while numerous different subclones with additional oncogenic alterations were found spread throughout the brain (2, 51, 65). These findings imply that DIPG cells leave the pons early during tumor evolution, before onset of aggressive expansion and the acquisition of multiple tumor subclones (51, 65). However, no correlation between specific driver mutations and dissemination sites has been established, whereas in adult GBM distinct tumor subpopulations, marked by amplification in differential genes, were shown to prefer certain niches, such as perivascular zones (64, 83).

### DIPG Follows Preferred Routes for Invasion

It seems that DIPG invasion within the central nervous system does not occur randomly, as some regions are more often invaded than others. Tumor cells were seen to have spread to the midbrain and medulla in 63% of cases, to the cerebellum and thalamus in 56%, and to the frontal cortex and supratentorial leptomeninges in 25% of cases (10). These findings suggest that DIPG cells follow preferential routes for dissemination along pre-existing structures, or are guided by signaling molecules from specific niches in order to invade distal sites, which is a well-established concept for cancer metastasis (84). Pericellular and axonal location of DIPG cells in patient-derived xenograph models that strongly recapitulate human disease (79), suggest that tumor cells use neural cell stands as railways to migrate through the brain. This trait could be reminiscent of neuroblasts that use radial glial fibers as migratory tracks to guide them during embryonic brain development (85, 86). GBM tumors have been shown to travel along white matter tracts (87) or to follow blood vessels (88). Although information on DIPG invasion routes is limited, perivascular spread has been observed in patient autopsy material (89). Considering the discrete temporal pattern of DIPG progression during brain development, it is conceivable that highly specific signals involved in brain development contribute to DIPG invasion, but this remains to be investigated (90). Moreover, DIPG's specific location plays a role as well with the pontine environment presenting a critical factor. This has been demonstrated by tumors carrying the same driver mutations, but originating in different anatomical locations than the pons, having a better prognosis (91).

### Specific Spread Into the Subventricular Zone

Interestingly, in 63% of patients, Caretti et al. noted invasion of the subventricular zone (SVZ) (10), which had not been well-recognized before. This preferential spread into the SVZ indicates that chemoattractants from this distant region can support DIPG invasion. The SVZ is occupied by proliferating neuron progenitor cells (92) and DIPG cells were shown to migrate toward these cells in a Matrigel invasion assay (93). The growth factor pleiotrophin (PTN) and three of its binding partners secreted by neuron progenitor cells acted as chemoattractants for DIPG cells in this model. PTN is involved in neuronal migration during development and has been implied in GBM invasion as well (94, 95). To demonstrate the necessity of PTN for SVZ invasion *in vivo*, shRNA mediated knockdown of PTN was performed in a DIPG xenograft model. This indeed resulted in reduced spread to the SVZ, establishing PTN as a critical neuron progenitor cell derived factor implicated in DIPG invasion (93) (Figure 2D), and further stressing the role of the brain microenvironment in this process. In this regard, it is again important to consider that the microenvironment and signaling molecules influencing DIPG are different from the adult brain, due to the temporal origin of DIPG in the still developing brain of pediatric patients (90).

Collectively, these findings indicate that DIPG spreads preferentially to certain locations within the central nervous system. However, with the exception of PTN induced migration into the SVZ, additional preferred dissemination routes and/or specific chemoattractants that play a role in this specific invasion pattern still need to be clarified. Here, the tumor's particular spatiotemporal origin in the pons during brain development should again be taking into account.

## Cellular Components in the Microenvironment That Can Influence Invasion

Next to neuron progenitor cells described above, other non-neoplastic cells in the tumor microenvironment can also regulate the complex process of tumor invasion. Although the exact microenvironment of DIPG has been poorly characterized, since DIPG originates and spreads in the central nervous system, neurons represent a significant cellular compartment within the tumor's direct environment. In addition, infiltration of immune cells adds to the complexity of the tumor microenvironment and its specific cellular composition at both primary and metastatic sites has been shown to impact progression of other types of cancer (96).

### Activated Neurons Impact DIPG Cell Behavior

In a xenograft model of pediatric cortical glioblastoma, activation of cortical neurons through optogenetic stimulation was shown to enhance tumor cell proliferation, and the same was true for patient-derived DIPG cells *in vitro* (69). These data demonstrate that activated neurons can influence tumor cell behavior in DIPG, which was achieved, at least partially, through secretion of NLGN3. Indeed, patient-derived DIPG cell lines exhibited growth stagnation when xenografted into the pons or frontal cortex of *Nlgn3* deficient mice. As described above, NLGN3 could contribute to an interconnected network of DIPG cells and synergy between tumor subclones via the induction of TMs (Figure 2B). In addition, since NLGN3 induced downstream PI3K/Akt signaling (69), a pathway linked to tumor invasion as described above, it is likely that secretion of NLGN3 also contributes to DIPG invasion by activating this pathway. Collectively, these findings identify two potential mechanisms by which activated neurons in the tumor microenvironment can contribute to DIPG invasion through secretion of NLGN3 (Figure 2E), which warrants further investigation.

Additionally, two recent reports provide evidence for the presence of bona fide AMPA receptor-dependent synapses between healthy neurons and TMs in adult GBM and DIPG (67, 97). These reports show that DIPG cells can express AMPA receptors and form post-synaptic regions able to confer electrical signals from nearby active neurons. Depolarization of tumor cells led to a rise of cytoplasmic calcium concentrations, which could spread throughout connected cells by TMs and which correlated with increased invasion and migration (67, 97). Together, these data indicate a growth factor independent way for neuronal activation to stimulate invasion in DIPG, through synaptic signals that are transmitted throughout the electrically active tumor network.

### DIPG Displays a Unique Immune Environment With Minimal Immune Activation

Immune cells can influence the behavior of tumor cells and in adult GBM it was shown that infiltrating immune cells stimulate tumor proliferation and invasion (98, 99). Immunohistochemical staining of postmortem DIPG tissue and flow cytometry analysis of primary DIPG tumor cells revealed the presence of a population of macrophages/microglia (100) (Figure 2F). Microglia are resident macrophages in the central nervous system and have also been described to be present in the GBM microenvironment (101). Microglia can facilitate cell migration (102), for instance by directly secreting matrix metalloproteinases that break down the extracellular matrix (103), or by promoting their secretion by tumor cells through the production of TGF-ß1 (104). The role of immune infiltration in DIPG invasion has not yet been studied. However, comparison of the immune composition between DIPG and adult GBM demonstrated fewer T cells and more microglia/macrophages in primary DIPG tissue. Whole transcriptome analysis of this microglia/macrophage population revealed a reduced inflammatory phenotype in DIPG compared to GBM, including limited expression of cytokines and chemokines. This also involved the absence of inflammatory cytokines and chemokines that are normally involved in recruiting other immune cells to the microenvironment (105). In line with these findings, Lieberman et al. show that in contrast to pediatric high-grade and low-grade glioma, DIPG tissue does not show enhanced T cell infiltration compared to adjacent non-tumor tissue. In addition, no increase in chemokines and inflammatory cytokines was observed (106). Collectively, these findings demonstrate minimal immune activation in the DIPG microenvironment in contrast to other types of brain tumors. How this unique immune environment affects DIPG's invasive behavior remains to be determined.

## Therapeutic Targeting of DIPG Invasion

Despite over 200 clinical trials, so far not a single drug has proven effective in prolonging survival in DIPG patients (12). This failure in DIPG treatment can be attributed to several factors. Most importantly, as mentioned before, although DIPG and GBM are genetically and molecularly distinct diseases (14), most DIPG trials have focused on drugs that were seen effective in GBM (13), overlooking the specifics of DIPG pathology. This also results from a lack in suitable experimental models for DIPG that take into account its specific spatiotemporal and often relatively slow progression in the developing brain (79). In part because of practical reasons, fast growing, high proliferative models are often used, which might not adequately reflect tumor behavior in patients. Furthermore, DIPG seems to exhibit intrinsic resistance to therapies, resulting from a relative lack of proliferating cells and its diffuse invasive growth that is potentially amplified by the presence of heterogeneous tumor subclones connected by a network of TMs (66, 68). Finally, due to the tumor's anatomical location another huge challenge in effective treatment for DIPG is the delivery of drugs to the tumor site, which involves crossing the blood-brain barrier (BBB). This represents an even bigger obstacle for DIPG compared to other brain cancers, as in many brain tumors the BBB is compromised by the formation of a disordered and highly permeable neovasculature. However, this is not the case for highly infiltrative tumors, like DIPG that use the existing brain vasculature with a largely intact BBB (107). Indeed, especially at diagnosis, DIPG shows limited to no contrast enhancement after intravenous delivery of gadolinium, as opposed to GBM tumors that harbor highly neovascular regions (108). Below, we describe therapies currently in clinical trials, as well as new agents still under pre-clinical evaluation that have the potential to target DIPG invasion and briefly discuss strategies for effective delivery across the BBB.

### Novel Therapeutics With the Potential to Target DIPG Invasion

#### Ongoing Clinical Trials

Several drugs that target aberrations due to driver mutations in H3K27M and PDGFRA, or P13K/Akt signaling, and could thereby influence DIPG invasion, are currently being tested in clinical trials. These include Panobinostat, a histone deacetylase inhibitor that partially rescues H3K27 trimethylation (Figure 1A), while at the same time inducing H3 acetylation, thus causing an overall shift in epigenetic gene regulation (109, 110). Administration of Panobinostat in orthotopic xenograft models of DIPG inhibits tumor growth and prolongs survival (109). Multiple clinical trials with this promising drug are currently recruiting (clinicaltrials.gov). Furthermore, a number of trials involving inhibitors of PDGFRA, such as Imatinib, Crenolanib, and Dasatinib are completed or underway (Figure 1D). Imatinib was studied in a phase I trial, but might increase the risk of intratumoral hemorrhage (111). In contrast, Crenolanib and Dasatinib were both well-tolerated in children (112, 113). Importantly, Dasatinib was shown to inhibit invasion in DIPG cell lines *in vitro* (114), demonstrating that inhibition of PDFGRA indeed has the potential to target DIPG invasion. An additional phase I trial with Dasatinib has recently been completed, while a phase II trial is still recruiting (clinicaltrials.gov). Finally, mammalian target of rapamycin (mTOR) inhibitors that target the P13K/Akt pathway include Sirolimus and Everolimus (Figures 1C,D) and multiple trials with these therapeutics are currently recruiting (clinicaltrials.gov). Therefore, several clinical trials are being initiated or currently ongoing with new therapeutic agents that have the potential to target DIPG invasion. With no results reported yet (clinicaltrials.gov), it will be highly intriguing to see the outcome of these trials, since the current treatment standard for DIPG does not address the highly invasive nature of this disease.

#### New Targets Under Pre-Clinical Evaluation

Other drugs that have the future potential of targeting DIPG invasion are still being investigated in pre-clinical *in vitro* and *in vivo* models. These include the β-catenin antagonist, ICG-001 that was shown to inhibit the migratory and invasive behavior of pediatric high-grade glioma cell lines *in vitro* (115). Similarly, the GSK-3 inhibitor LiCL (Figure 1E), and the indirubin derivative BIO inhibited migration and spheroid invasion of two pediatric high-grade glioma cell lines, as well as a DIPG cell line (63). Furthermore, evidence for an interconnected brain-DIPG network containing AMPA-receptor dependent synapses warrants investigation into modulators of synaptic transmission, such as the AMPA receptor antagonist perampanel, which inhibits proliferation of DIPG cells in mice (67, 97). A recent report elucidates another therapeutic vulnerability in H3K27M tumors: increased amounts of acetylation marks lead to epigenetic dysregulation and immune invasion. These findings suggest the possible use of a combination of epigenetic and immune modulating therapies (116). Finally, the selective inhibitor of EPH receptor signaling, LDN-211904 (Figure 1E) also reduces spheroid invasion by patient-derived DIPG cell lines (60). Although the data still await full publication, a meeting abstract reports the effectiveness of the STAT3 inhibitor, WP1066 in inhibiting migration of DIPG primary cells, as well as reducing tumor burden and enhancing survival in a xenograft DIPG model (57). Other pre-clinical targets that have the potential of inhibiting DIPG invasion, include EZH2 inhibitors that would target the remaining H3K27me3 peaks in DIPG (117, 118) and newly developed ALK2/BMP type 1 receptor kinase inhibitory agents that would limit the impact of the ACVR1 constitutively activating mutations observed in DIPG (119). Although these preclinical advancements look highly promising, it remains to be seen whether these findings translate to patients and whether the investigated drugs can indeed effectively be used for treatment with minimal toxicity.

### Overcoming the Blood-Brain Barrier for Effective Therapeutic Targeting

One critical factor for whether newly developed therapeutic agents end up in clinical practice, depends on their ability to reach the central nervous system. Traversing the BBB is crucial for the efficacy of any potential DIPG treatment. This can be accomplished through various strategies, including the use of small molecule drugs able to cross the BBB based on their inherent properties (120). Additionally, permeabilization of the BBB can be increased through compounds, such as morphine or methamphetamine (121–123). Furthermore, convection enhanced delivery (CED) makes it possible to distribute drugs directly into the tumor microenvironment via a highly controlled positive pressure gradient and a constant flow induced by a pump (124). Importantly, these CED systems have been proven safe and effective to use in DIPG (125). Another promising new technology, feasible although not yet applied in DIPG, is microbubble and focused ultrasound-mediated BBB disruption (126). This technique utilizes ultrasonic waves in combination with administrated microbubbles to permeabilize the capillary bed of the BBB. Whichever drugs will be applied for the future treatment of DIPG invasion, it will be crucial to select them based on their brain penetration properties or combine them with one of these BBB traversing strategies to effectively deliver them to the tumor.

## Conclusion and Future Perspective

DIPG remains a universally lethal disease with no hope for recovery. An important cause for this dire prognosis is the diffuse infiltrative phenotype of the tumor, which together with its anatomical location, greatly restrict the effectiveness of conventional treatment methods. The process of DIPG invasion has been very poorly investigated, although the amount of studies on DIPG is rapidly increasing, due to advancements in both experimental techniques and clinical procedures. This has provided novel mechanistic insights and revealed multiple intrinsic and extrinsic factors involved in disease progression. Although our understanding of the exact underlying mechanisms and the interplay between these different factors is still limited, some of them can now be connected. All of the most recurring mutations in DIPG can be linked to pathways that activate mesenchymal-related transcription factors. Although EMT presents a well-known concept of cancer progression by promoting migration and metastasis, the extent to which mesenchymal transition contributes to DIPG invasion still requires future investigation.

To fill in the many gaps in our understanding of the invasive spread of DIPG, more research is urgently needed. Going forward, it will be especially important to investigate and counteract DIPG invasion *in vivo*, as most of our knowledge on potential factors involved in DIPG invasive cell behavior still comes from *in vitro* models. Evidently, these culture models lack important cellular context that is present *in vivo*, including the tumor microenvironment that has such a strong impact on tumor cell behavior (127). This is even more relevant in the context of DIPG, given its unique origin in the pons during brain development (90). DIPG's spatiotemporal progression will therefore encounter very unique environmental conditions and signals that can have a specific impact on tumor cell behavior. As such, it will be key to develop relevant DIPG *in vivo* models that mimic the spatiotemporal origin of DIPG. Ideally these models should involve tumor induction locally in the pons early during brain development and account for the often relatively slow progression of tumors observed in patients. In addition, experimental DIPG models should recapitulate the different driver mutations and subclasses of disease that are now recognized in patients (16). Therefore, *in utero* electroporation models provide an optimal approach by enabling both disease onset in the developing brain and the introduction of specific disease associated driver mutations (59).

In these relevant *in vivo* models, invasion should be analyzed during tumor progression and here the recent recognition of DIPG heterogeneity with multiple tumor subclones cooperating to induce invasive behavior should be considered (65). Although bulk sequencing analyses have provided key insight into the underlying genetic and molecular biology of DIPG (16), single cell technologies will be the next step forward to take into account this inter-tumor heterogeneity (27). Next to genomic and transcriptomic approaches, high-resolution imaging will be of specific benefit for studying dynamic invasive behavior at the single-cell level. Intravital imaging, the visualization of single cells in living tissues through imaging windows (128), together with optogenetic tools will allow to characterize the invasive behavior of single cells over time (129, 130). As such, insights can be obtained regarding the heterogeneity of invasive tumor cell populations. In addition, recent advances in high volume imaging now make it possible to visualize entire tumors and their surrounding tissue at high resolution (131–135) and should therefore be implemented to confirm knowledge on invasive behavior from animal models in human autopsy material. Next to translating findings from mice to human, these datasets can reveal the impact of invasive tumor cell subsets for clinical outcome, by correlating the presence of invasive cell populations to disease course. With these technologies it will also be possible to unambiguously confirm the presence of TMs (68) and EVs (72) in DIPG and shed light on their involvement in tumor invasion by analyzing their presence in highly invasive compared to static tumor regions. Ultimately these future research goals directed at deciphering DIPG's highly invasive nature might lead to the discovery of new therapeutic strategies that could finally satisfy the urgent need for an effective therapy.

## Author Contributions

TK and MA performed the literature review and wrote the manuscript. EW and AR provided supervision, revised, and edited the manuscript. DV provided critical expertise and feedback and helped with conceptualization of the manuscript.

### Conflict of Interest

The authors declare that the research was conducted in the absence of any commercial or financial relationships that could be construed as a potential conflict of interest.
